# Outpatient Dispensing Patterns and Changes in Tirzepatide Strength Mix in Japan: A Study Using the National Open Claims Data

**DOI:** 10.7759/cureus.113681

**Published:** 2026-07-30

**Authors:** Takuya Omura, Takahiro Kamihara

**Affiliations:** 1 Department of Metabolic Research, National Center for Geriatrics and Gerontology, Obu, JPN; 2 Department of Diabetes and Endocrinology, National Center for Geriatrics and Gerontology, Obu, JPN; 3 Department of Cardiology, National Center for Geriatrics and Gerontology, Obu, JPN

**Keywords:** claims data, dispensing setting, drug utilization, pharmacoepidemiology, tirzepatide, type 2 diabetes

## Abstract

Background: Tirzepatide is a once-weekly injectable dual agonist of the glucose-dependent insulinotropic polypeptide (GIP) and glucagon-like peptide-1 (GLP-1) receptors; in Japan, it is approved for the treatment of type 2 diabetes and marketed under the brand name Mounjaro, which is available in six strengths. Japanese studies of tirzepatide have primarily evaluated efficacy, real-world effectiveness, safety, persistence, and patient-level maintenance doses. National patterns in dispensing settings and longitudinal changes in the product-strength mix have not been described. We examined whether increases in claim-recorded quantities were accompanied by structural changes in outpatient dispensing and strength mix.

Methods: We analyzed injectable-drug quantity tables for the fiscal year 2024 from the 11th National Database of Health Insurance Claims and Specific Health Checkups of Japan Open Data. Full National Health Insurance drug codes were used to identify all six Mounjaro strengths. The primary tables excluded public-expense claims, and public-expense-inclusive tables were used in a sensitivity analysis. Monthly analyses required numeric values for every strength in both the within-institution and outside-institution outpatient prescription worksheets. Outcomes included annual quantities by dispensing category and strength, monthly total quantity, the share dispensed outside medical institutions, strength-mix indicators, and each strength's contribution to the increase from June 2024 to March 2025.

Results: The primary outpatient total was 7,829,974.7 kit units. Of this total, 6,748,743.5 kit units (86.2%) were recorded as outpatient prescriptions dispensed outside medical institutions. Complete monthly data were available from June 2024 through March 2025. During this interval, monthly quantity increased from 392,354 to 1,027,955 kit units; outside-institution quantity increased from 329,431 kit units (84.0%) to 899,994 kit units (87.6%); and the quantity represented by strengths of 7.5 mg or higher increased from 50,902 kit units (13.0%) to 246,316 kit units (24.0%). The quantity of the 2.5-mg product increased from 159,318 kit units (40.6%) to 278,540 kit units (27.1%), while its share decreased. The 5-mg product accounted for 320,965 kit units (50.5%) of the absolute increase, whereas strengths of 7.5 mg or higher collectively accounted for 195,414 kit units (30.7%). The public-expense-inclusive sensitivity analysis preserved the ranking of strengths and the predominance of outside-institution dispensing.

Conclusions: Tirzepatide dispensing in Japan was concentrated in the outside-institution outpatient category, and the share in this category increased over the fully observable period. Growth was also accompanied by a measurable shift in the product-strength mix. This study provides an auditable national description of dispensing-setting and product-mix dynamics rather than an analysis of patient uptake, adherence, clinical outcomes, or individual titration.

## Introduction

Japanese phase 3 trials demonstrated the efficacy of tirzepatide for glycemic control and body-weight reduction across dose regimens [1,2]. Tirzepatide is a once-weekly dual agonist of the glucose-dependent insulinotropic polypeptide and glucagon-like peptide-1 receptors and is approved in Japan for type 2 diabetes [3]. The Japanese electronic package insert specifies a starting dose of 2.5 mg, a maintenance dose of 5 mg, optional 2.5 mg increments at intervals of at least four weeks as needed, and a maximum weekly dose of 15 mg [3]. The 2.5 mg and 5 mg Mounjaro products were launched on April 18, 2023 [4], followed by the 7.5, 10, 12.5, and 15 mg products on June 12, 2023 [5].

Following the launch, the Japanese evidence base expanded from phase 3 trials to include an observational pilot study of continuous glucose monitoring in older patients and clinic-based real-world cohorts evaluating glycemic and weight outcomes, safety, persistence, maintenance-dose distributions, and outcomes among patients without obesity [6-11]. These studies provide valuable patient-level clinical context but do not describe dispensing settings or the aggregate product-strength mix at the national level. National open claims data address a complementary health-services question: not whether tirzepatide is effective in selected patients, but how reimbursed product quantities are distributed across outpatient dispensing categories and strengths nationwide.

We therefore examined nationwide outpatient quantities during fiscal year 2024, the first complete Japanese fiscal year after all six strengths became available. The structural questions were whether dispensing was concentrated in the outside-institution category, whether the strength composition changed during market expansion, and which strengths accounted for the absolute increase between the first and last months with complete data. No causal or patient-level inference was intended.

## Materials and methods

Study design and data source

We conducted a retrospective descriptive analysis of publicly available aggregate data from the 11th National Database of Health Insurance Claims and Specific Health Checkups of Japan (NDB) open data. The study period extended from April 1, 2024, through March 31, 2025 [12]. The primary workbooks excluded public-expense claims; corresponding public-expense-inclusive workbooks were examined in a sensitivity analysis. Because the data comprised published aggregate quantities rather than individual records or a statistical sample, we did not calculate confidence intervals or perform hypothesis tests. The study was submitted to the Ethics and Conflict of Interest Committee of the National Center for Geriatrics and Gerontology to determine whether ethical review was required. On June 29, 2026, the committee chair determined that ethical review was not applicable. As the study used only publicly available aggregate data without identifiable individual-level information, informed consent was not applicable.

Literature context search

To assess whether this study duplicated existing Japanese tirzepatide research, we conducted targeted searches of PubMed and publisher websites through July 13, 2026. Search terms combined "tirzepatide" with "Japan," "real-world," "claims," "utilization," "dispensing," "product strength," "NDB open data," "older adults," "body mass index," and "non-obesity." We also screened reference lists and related-article links for additional Japanese studies. This contextual search was not intended as a systematic review or meta-analysis.

Product identification and care settings

Mounjaro products were identified using the full National Health Insurance drug codes 2499422G1024, 2499422G2020, 2499422G3027, 2499422G4023, 2499422G5020, and 2499422G6026 for the 2.5, 5, 7.5, 10, 12.5, and 15 mg products, respectively [3,12]. Each code appeared once in each relevant worksheet, and the source unit was 'kit.' We analyzed worksheets for outpatient prescriptions dispensed within and outside medical institutions; inpatient quantities were examined separately and were not combined with outpatient quantities.

Masking and calculation rules

Hyphenated cells were treated as unknown suppressed values. We did not assign them a value of zero, 399, or any other upper bound, and we did not impute them [12]. Numeric zeros were retained. Annual outpatient quantities were calculated as the arithmetic sum of the underlying numeric values in the within- and outside-institution outpatient worksheets. Monthly analyses across all strengths were limited to June 2024 through March 2025 because the within-institution 15 mg cells for April and May 2024 were suppressed. For each month with complete data, 5 mg quantities were read directly from the 5 mg NDB rows and summed across the two outpatient dispensing settings; they were not calculated as a residual. Quantities for strengths of 7.5 mg or higher were calculated by summing the 7.5, 10, 12.5, and 15 mg source rows.

Outcome measures

The primary measures were annual outpatient quantity by strength and dispensing setting; the proportion of annual outpatient quantity dispensed outside institutions; monthly total outpatient quantity; monthly outside-institution share; and monthly strength composition in three mutually exclusive groups (2.5 mg, 5 mg, and ≥7.5 mg). We also calculated the exact kit units and the corresponding percentage values for the 2.5 mg and ≥7.5 mg groups and an exploratory kit-weighted mean labeled strength. The latter was a composition index based on labeled product strengths and kit quantities, not a measure of prescribed dose, exposure, or patient-level titration.

Statistical analysis

All analyses were descriptive. Annual and monthly quantities, dispensing-setting and strength proportions, the descriptive March-to-June ratio, each strength group's contribution to the absolute change between June 2024 and March 2025, and the kit-weighted mean labeled strength were calculated directly from the underlying numeric source-cell values using Microsoft Excel 2019 (Microsoft Corporation, Redmond, WA, USA). No inferential tests were performed. Unrounded values were retained for all calculations; percentages and the weighted mean were rounded only for presentation.

## Results

Annual outpatient distribution

The primary outpatient total was 7,829,974.7 kit units. Of this total, 6,748,743.5 kit units (86.2%) were recorded in the outside-institution dispensing category and 1,081,231.2 kit units (13.8%) in the within-institution category. Among individual strengths, the outside-institution share was lowest for the 15 mg product (83.2%) and, after rounding to one decimal place, was 88.2% for both the 7.5 and 10 mg products. The annual distribution by strength was 2,375,269.5 kit units (30.3%) for 2.5 mg; 3,919,581 kit units (50.1%) for 5 mg; 998,672.5 kit units (12.8%) for 7.5 mg, 354,297.5 kit units (4.5%) for 10 mg; 104,508.2 kit units (1.3%) for 12.5 mg; and 77,646 kit units (1.0%) for 15 mg (Table 1). The public-expense-inclusive outpatient total was 8,345,367.2 kit units, which was 515,392.5 kit units (6.6%) higher than the primary total. Because no denominator for the eligible population was available, these quantities should not be interpreted as prevalence of use.

**Table 1:** Annual outpatient Mounjaro quantities by strength and dispensing setting in fiscal year 2024 (April 2024 through March 2025) NHI: National Health Insurance. The primary tables exclude public-expense claims. Values are kit quantities from the underlying source cells; the source workbook displays numeric cells rounded to whole units. Inpatient quantities are not included.

Strength	Full NHI drug code	Within institution	Outside institution	Outpatient total	Share of primary total	Public-expense-inclusive total
2.5 mg	2499422G1024	337,412.50	2,037,857	2,375,269.50	30.30%	2,528,775.50
5 mg	2499422G2020	556,342	3,363,239	3,919,581	50.10%	4,159,477
7.5 mg	2499422G3027	117,500	881,172.50	998,672.50	12.80%	1,069,596.50
10 mg	2499422G4023	41,679.50	312,618	354,297.50	4.50%	385,045
12.5 mg	2499422G5020	15,249.20	89,259	104,508.20	1.30%	115,012.20
15 mg	2499422G6026	13,048	64,598	77,646	1.00%	87,461
Total	-	1,081,231.20	6,748,743.50	7,829,974.70	100.00%	8,345,367.20

Monthly quantity and strength-mix evolution

**Figure 1:**
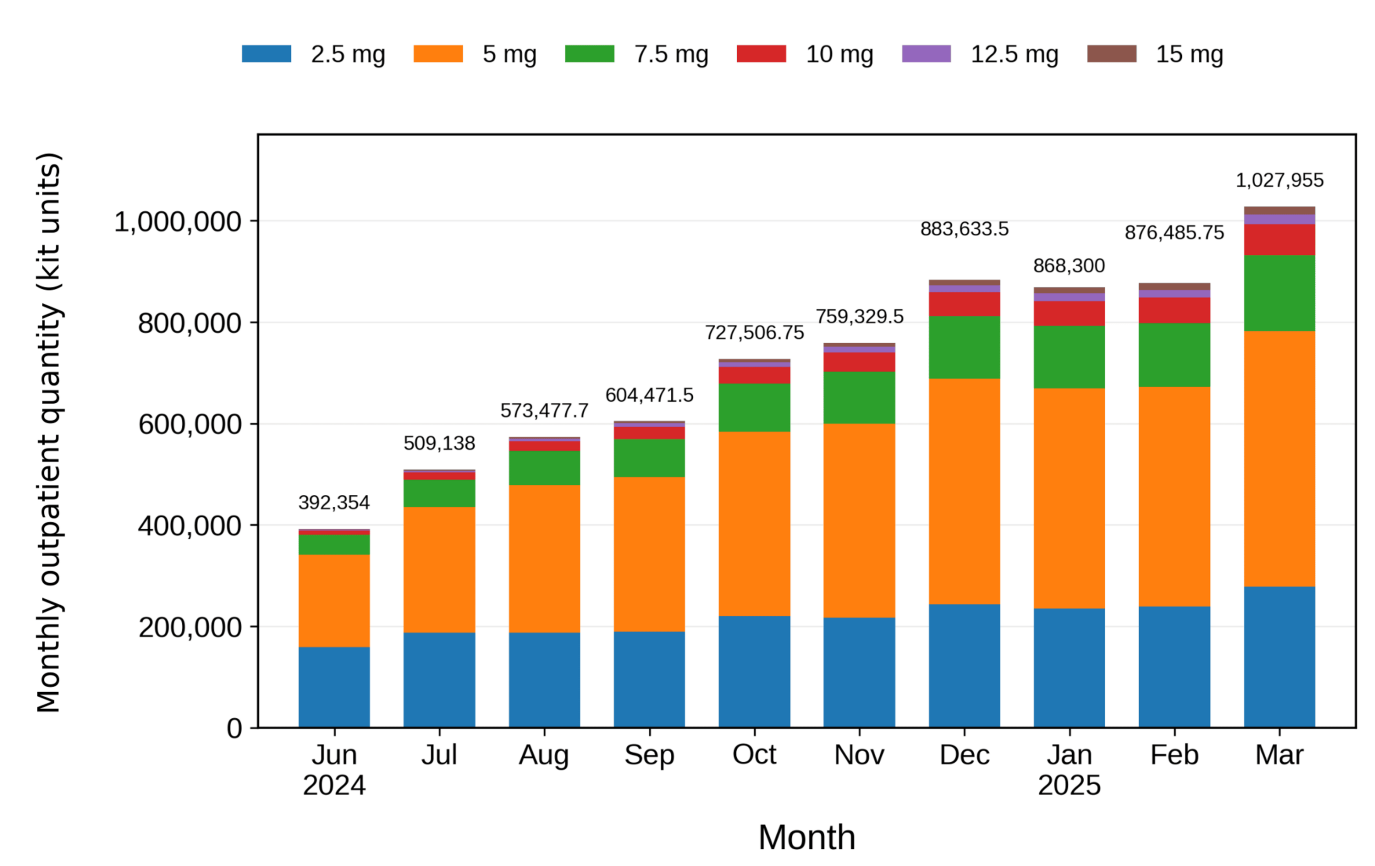
Monthly outpatient Mounjaro quantities by individual product strength, June 2024 through March 2025 The stacked bars represent exact kit quantities for all six labeled strengths. Within- and outside-institution outpatient quantities were summed from the underlying numeric source-cell values. April and May are omitted because the within-institution 15 mg cells were suppressed. The primary tables exclude public-expense claims, and inpatient quantities are not included. Labels above the bars show exact monthly totals, including decimal quantities when present in the source-derived sums.

The within-institution 15 mg cells were suppressed in April and May 2024; exact all-strength outpatient totals and dispensing-setting shares were therefore not calculated for those months. For each month from June 2024 through March 2025, all 12 component cells contained numeric values. During this period, monthly quantity increased from 392,354 to 1,027,955 kit units, corresponding to a descriptive March-to-June ratio of 2.62 (Figure 1), while the outside-institution quantity increased from 329,431 kit units (84.0%) to 899,994 kit units (87.6%). Monthly quantity did not increase monotonically: it declined in January, partially recovered in February but remained below the December level, and then increased markedly in March.

**Figure 2:**
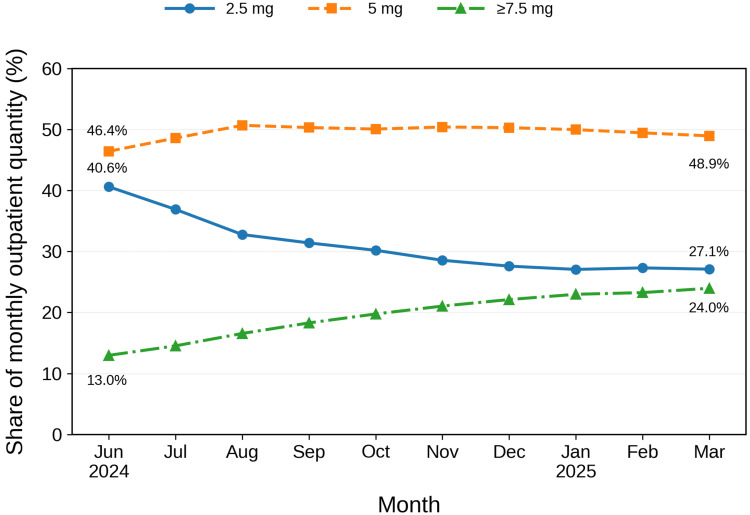
Monthly outpatient Mounjaro strength composition, June 2024 through March 2025 The lines show three mutually exclusive labeled-strength groups: 2.5 mg, 5 mg, and ≥7.5 mg (7.5, 10, 12.5, and 15 mg combined). The 5 mg series was obtained directly from the NDB rows for NHI drug code 2499422G2020 and summed across the within- and outside-institution outpatient worksheets; it was not calculated as a residual. The three groups sum to 100% in each month. The primary tables exclude public-expense claims; inpatient quantities are not included. April and May are omitted because the within-institution 15 mg cells were suppressed. NDB: National Database of Health Insurance Claims and Specific Health Checkups of Japan, NHI: National Health Insurance

Figure 1 shows absolute monthly quantities for all six individual strengths. Figure 2 shows the complete monthly composition in three mutually exclusive groups (2.5 mg, 5 mg, and ≥7.5 mg), whereas Table 2 reports exact kit units and the corresponding percentage for selected groups and the kit-weighted mean labeled strength. From June 2024 to March 2025, the quantity of the 2.5 mg product increased from 159,318 kit units (40.6%) to 278,540 kit units (27.1%), despite a decrease in its share. The directly observed 5 mg quantity increased from 182,134 kit units (46.4%) to 503,099 kit units (48.9%), while the quantity represented by strengths of 7.5 mg or higher increased from 50,902 kit units (13.0%) to 246,316 kit units (24.0%) (Figure 2; Table 2). The kit-weighted mean labeled strength increased from 4.42 to 5.28 mg per kit (Table 2). Total quantity increased by 635,601 kit units: the 5 mg product accounted for 320,965 kit units (50.5%) of this increase, the 2.5 mg product accounted for 119,222 kit units (18.8%), and strengths of 7.5 mg or higher collectively accounted for 195,414 kit units (30.7%).

**Table 2:** Monthly outpatient quantity and selected strength-mix indicators from June 2024 through March 2025 The table reports selected indicators, whereas Figure 2 shows the complete three-group composition. Monthly quantities exclude public-expense claims and inpatient quantities. Analyses were limited to June 2024 through March 2025 because the within-institution 15 mg cells for April and May 2024 were suppressed. The kit-weighted mean labeled strength is an exploratory composition measure calculated from labeled product strengths and kit quantities; it is not a measure of prescribed dose, exposure, or patient-level titration. The kit units are exact sums of the underlying within- and outside-institution source cells. Percentages were calculated from exact kit units and rounded to one decimal place; therefore, kit units should not be reconstructed from the displayed percentages.

Month	Total kit units	Strengths ≥7.5 mg, kit units (%)	2.5 mg, kit units (%)	Kit-weighted mean labeled strength (mg per kit)
Jun 2024	392,354	50,902 (13.0)	159,318 (40.6)	4.42
Jul 2024	509,138	73,983 (14.5)	187,818 (36.9)	4.58
Aug 2024	573,477.7	94,965.7 (16.6)	187,937 (32.8)	4.76
Sep 2024	604,471.5	110,545.5 (18.3)	189,802 (31.4)	4.88
Oct 2024	727,506.75	143,718.5 (19.8)	219,628.25 (30.2)	4.98
Nov 2024	759,329.5	159,820.5 (21.0)	216,815 (28.6)	5.09
Dec 2024	883,633.5	195,438.5 (22.1)	243,824 (27.6)	5.17
Jan 2025	868,300	199,527 (23.0)	234,930 (27.1)	5.23
Feb 2025	876,485.75	203,826.5 (23.3)	239,338.25 (27.3)	5.24
Mar 2025	1,027,955	246,316 (24.0)	278,540 (27.1)	5.28

Inpatient and sensitivity findings

Complete inpatient totals could not be calculated because the annual 12.5 and 15 mg inpatient cells were suppressed. In the outpatient sensitivity analysis, inclusion of public-expense claims increased the total quantity by 515,392.5 kit units but did not materially change the ranking of strengths.

## Discussion

This national analysis identified a clear outpatient dispensing pattern. A total of 6,748,743.5 kit units (86.2%) were recorded in the category for outpatient prescriptions dispensed outside medical institutions, and the share in this category increased over the fully observable period. These findings describe how quantities were categorized in the claims data; this category does not establish home administration, self-injection, community-pharmacy market share, or the site of treatment decisions.

Growth in total quantity was accompanied by a shift in the strength mix. The 5 mg presentation accounted for 320,965 kit units (50.5%) of the absolute increase, while strengths of 7.5 mg or higher accounted for 195,414 kit units (30.7%) and increased their collective share. The quantity of the 2.5 mg presentation increased from 159,318 kit units (40.6%) to 278,540 kit units (27.1%), although its share decreased. Thus, total kit quantities increased over the study period while the product mix shifted toward higher labeled strengths. These presentation-level quantities do not demonstrate individual titration or dose escalation.

These findings complement rather than duplicate Japanese patient-level studies. An observational pilot study reported changes in continuous glucose monitoring metrics in older patients, and clinic-based cohorts have reported glycemic and weight responses, safety, persistence, maintenance-dose distributions, and outcomes among patients without obesity [6-11]. The units of analysis in those studies were individual patients at selected institutions, whereas the present study used national claim-recorded kit quantities without individual-level linkage. Our targeted search did not identify a peer-reviewed nationwide analysis that jointly reported dispensing setting, longitudinal strength composition, and strength-specific contributions to growth. Data from the NDB Open Data initiative have previously supported nationwide descriptive analyses of drug utilization and postmarketing safety in other therapeutic areas, including antihypertensive agents, anti-HIV therapy, and regulatory safety-signal assessment [13-15]. This prior use supports the suitability of the data source for the present descriptive purpose. The novelty of this study therefore lies in health-services and supply-structure surveillance, not in the evaluation of clinical effectiveness or individual titration.

Japan is also a clinically important setting for patient-level research because many treated patients with type 2 diabetes are older, and not all have obesity. The pilot continuous glucose monitoring study included four older Japanese patients with a mean age of 79.5 years and a mean body mass index (BMI) of 24.6 kg/m² [6]. A Hokkaido-TZP subanalysis identified 107 Japanese patients with a BMI below 25.0 kg/m²; 100 were included in the efficacy analysis, and reductions in glycated hemoglobin (HbA1c) were observed across baseline BMI tertiles over six months [10]. The broader clinical context is illustrated by a Japan Diabetes Comprehensive database project based on an Advanced Electronic Medical record System (J-DREAMS) analysis of 10,151 patients treated at Japanese referral centers, in which the mean age was 66.0 years [16]. Although the J-DREAMS cohort is not nationally representative, these studies illustrate the relevance of age and adiposity in Japan. Although aggregate sex- and age-stratified prescription-quantity tables are available in the NDB Open Data, these strata were not analyzed in the present study. The aggregate data do not permit identification of individual older adults or classification by obesity status, but the present findings provide population-level context for interpreting and extending this emerging patient-level evidence.

Several mechanisms could underlie this redistribution. The labeled regimen begins at 2.5 mg weekly, increases to 5 mg after four weeks, and permits additional 2.5 mg increments at intervals of at least four weeks when clinically appropriate [3]. The aggregate pattern may reflect individual titration, treatment initiation, discontinuation, refill timing, stock conditions, and prescribing preferences. Because the open tables do not link dispensing records at the individual level, these mechanisms cannot be distinguished.

The public-expense-inclusive analysis yielded an outpatient quantity that was 515,392.5 kit units (6.6%) higher than the primary estimate while preserving the ranking of strengths and the predominance of outside-institution dispensing. This finding supports the robustness of the structural results, although absolute quantities depend on whether public-expense claims are included. The analysis also highlights the importance of retaining exact decimal values and handling suppressed cells conservatively: treating the April and May 15 mg cells as zero would have produced spuriously precise monthly totals.

This study has several limitations. The open data report aggregate quantities rather than unique patients, prescriptions, or administrations. No population denominator was available, precluding estimates of prevalence or incidence. Although aggregate sex- and age-stratified prescription-quantity tables are available, these strata were not analyzed in the present study. The open data do not permit patient-level linkage of product quantities to age, sex, BMI, HbA1c, concomitant therapy, adherence, clinical outcomes, persistence, or reasons for changing strength, and self-pay use is not captured. Exact all-strength outpatient monthly totals were unavailable for April and May because the within-institution 15 mg cells were suppressed; inpatient totals were incomplete, and no comparator drug or previous fiscal year was analyzed. The kit-weighted mean labeled strength is an exploratory composition measure, not a dose measure. The literature search was targeted rather than systematic, and the absence of an identified national study does not prove that none exists. Finally, the observed growth and redistribution cannot be attributed to any particular clinical, commercial, or policy-related cause.

For health-services surveillance, this analysis distinguishes growth in total claim-recorded quantity from redistribution across product strengths. The persistent predominance of the outside-institution category identifies the principal outpatient dispensing category represented in the data and may provide useful descriptive information for strength-specific supply planning. Because suppressed cells were not imputed and every reported monthly indicator required complete observation, the estimates provide a transparent benchmark for comparison with future NDB Open Data releases. Future NDB Open Data releases, population denominators, and comparator therapies will be needed to determine whether the observed dispensing structure persists and whether it is specific to tirzepatide. Linkage to patient-level clinical data would be necessary to evaluate individual titration, adherence, effectiveness, and safety.

## Conclusions

National claims data for fiscal year 2024 showed that outpatient tirzepatide dispensing was concentrated in the category for prescriptions dispensed outside medical institutions and that the share in this category increased during the fully observable monthly period. Growth in total kit quantity was accompanied by a shift in composition away from the 2.5 mg presentation and toward higher-strength presentations, while the 5 mg product remained the largest contributor to absolute growth. These findings provide an auditable benchmark for health-services surveillance and may inform future strength-specific supply assessments; they do not estimate patient uptake or clinical effectiveness. Patient-linked data, population denominators, comparator products, and data from additional fiscal years are needed to determine the clinical drivers and persistence of the observed patterns.
